# Age structure of cohorts of mosquitoes from the field using shortwave infrared spectroscopy before and after ULV adulticide treatment

**DOI:** 10.1186/s13071-025-06873-1

**Published:** 2025-07-01

**Authors:** Christopher L. Swab, Barry W. Alto, Georgette Kluiters, Frank H. Cornine, Sam R. Telford

**Affiliations:** 1 Department of Infectious Disease and Global Health, Cummings School of Veterinary Medicine, Tufts University, North Grafton, MA USA; 2 Florida Medical Entomology Laboratory, Institute of Food and Agricultural Sciences, University of Florida, Vero Beach, FL USA; 3 Entomology and Nematology Department, University of Florida, Gainesville, FL USA; 4 Central Massachusetts Mosquito Control Project, Northborough, MA USA

**Keywords:** Mosquito, Spectroscopy, Age-structure, Risk assessment, Insecticide

## Abstract

**Background:**

The timely assessment of mosquito control efficacy through monitoring the age structure of wild cohorts of adult mosquitoes would improve operational decision making by control personnel. Analysis of shortwave infrared cuticular spectra for cohorts of laboratory reared *Anopheles gambiae* and *Aedes aegypti* of known age has shown that spectra outlier fraction is higher for cohorts of younger versus older weighted average age. This study investigates differences in outlier fraction of shortwave infrared cuticular spectra from wild cohorts of host-seeking mosquitoes of different species collected pre- and postultra-low volume (ULV) adulticiding (pyrethroid derivative, Etofenprox), with the hypothesis that post-treatment cohort spectra will have a higher outlier fraction than pretreatment due to younger mosquitoes replacing older ones killed by treatment.

**Methods:**

Over 15,000 mosquitoes representing eleven species were collected 1 day pre-ULV adulticide application and 2 days post application during four biweekly treatments conducted at a site near Westford, Massachusetts from July to August of 2023. Shortwave infrared absorbance measurements were taken on 3100 specimens apportioned from all treatments and collection days, and spectra were then aggregated to pre- and post-treatment datasets for each species.

**Results:**

Measurable changes occurred in pre- versus post-treatment cohort spectra outlier fraction for all species. A total of 8 of 11 species showed an increase in outlier fraction for post-treatment cohorts when aggregated over a 2-day post-treatment period, indicating replacement of older by younger mosquitoes. Analysis of abundance versus spectra outlier fraction over pre- and post-treatment collection days showed varying trends by species, implying an impact from recruitment of adults from new cohorts during the post-treatment period.

**Conclusions:**

We believe the technique shows promise for monitoring the age-structure of wild cohorts of mosquitoes over time. The method is particularly suitable for surveillance programs since it is rapid, incorporates economic equipment, involves only minimal training, does not require freshly killed mosquitoes and does not use machine learning. Future research should comprise longer post-treatment periods for better trend analysis and be directed toward geographically distinct and problematic mosquito vectors of importance. Further refinements in assessing the utility of the outlier fraction technique for age-grading may consider the influence of mosquito diet and infection with pathogens and focus on potential impacts from mosquito diet and parasitism.

**Graphical abstract:**

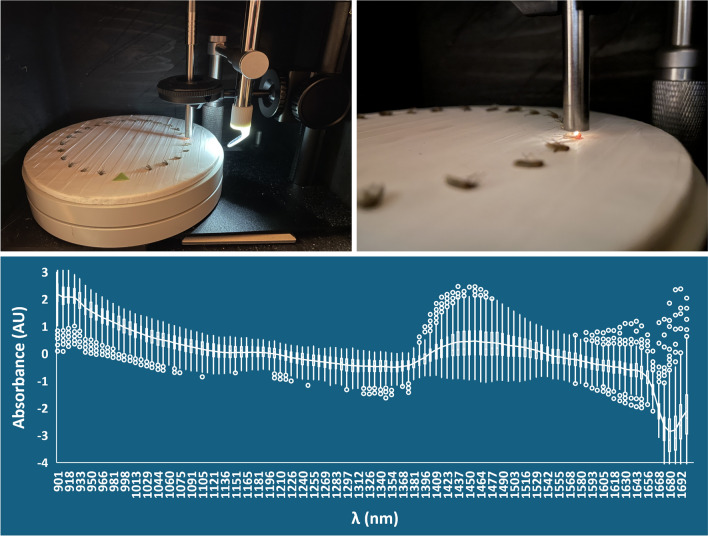

**Supplementary Information:**

The online version contains supplementary material available at 10.1186/s13071-025-06873-1.

## Background

Accurately quantifying the age structure of wild mosquito populations would prove beneficial to the assessment of vector dynamics and epidemiological trends, since mosquito age is a critical factor in the transmission of pathogens. Mosquito age is strongly associated with vector competence, as the extrinsic incubation period (EIP) (e.g., time measured in days for 50% of mosquitoes to become capable of transmitting the pathogen) must be complete before a mosquito becomes “infectious” and can transmit the pathogen. With rare exceptions, mosquitoes must feed on an infected host before being able to transmit pathogens. Younger mosquitoes are less likely to have fed and completed the EIP, thus reducing their contribution to the enzootic cycle or to public health risk. Both vector competence (ability to transmit) and the length of time that mosquitoes in a population have been alive (probability of survival) are key components of vectorial capacity [1], and as such, the age-grading of wild mosquitoes is of particular interest to mosquito control professionals and medical entomologists for determining the potential for epidemiological impacts. Age grading would provide more information about the efficacy of adulticiding than the typical pre- and post-spray measures of abundance inasmuch as newly emerged mosquitoes could confound such an analysis.

Ovary dissection is the current benchmark of gauging mosquito age and has been used to demonstrate the impact of ULV adulticiding on the age structure of populations of wild *Culex pipiens* and *Culex restuans* [2, 3]; however, it has significant drawbacks in that it requires freshly killed mosquitoes and can be time consuming, which can make it challenging for use in seasonal surveillance programs or clinical research. Digital image analysis of wing wear shows promise as an age grading method [4], although mosquitoes sampled by standard collection methods can be affected by loss of scales and other morphological alterations. The use of infrared spectroscopy (IRS) to analyze the mosquito cuticle may prove to be as accurate for age-grading wild populations of mosquitoes as ovary dissection with the added benefits of being less labor intensive and more versatile, rapid, and straightforward.

Most prior studies using IRS for age grading mosquitoes have relied upon metrics developed from and tested on cohorts of laboratory reared specimens of known age, with research conducted in the near infrared (NIRS) [4–17] and mid-infrared (MIRS) [18–22] regions. In addition to mosquitoes reared entirely in the laboratory from eggs, some studies have included cohorts raised under field-like conditions [6, 8, 16] or from field-collected larvae or pupae [10, 13, 17]. The chemometric used most frequently for age prediction is regression modeling [5–15, 17–20, 22], but artificial neural networks (ANN) [16], and deep and transfer learning methodologies combined with spectra dimensionality reduction [21] have also been employed. Research has been concentrated on sub-Saharan African malarial vectors [5–10, 13, 14, 16, 19–22] as well as *Aedes aegypti* [11, 12, 18] and *Aedes albopictus* [15, 17]. Prediction accuracy for these studies typically ranges from 70% to greater than 95%.

IRS for age determination of wild-caught mosquitoes has been less successful when incorporating regression modeling but has achieved high accuracy with the aid of more advanced chemometrics. NIRS coupled with regression modeling has assigned cohort ages of wild *Anopheles gambiae* s.l. that correspond to mean chronological ages based on ovary dissection results [6] and shown that the modeled age distributions of wild *Anopheles* spp. are similar for insecticide-susceptible and resistant populations [10]. The technique has been used to designate the age of wild *Anopheles gambiae* and *Anopheles arabiensis* with approximately 67–69% accuracy [13, 23] but has also been found to be unsuitable for modeling the precise age of wild *Ae. aegypti* [24]. However, NIRS and ANN models trained from and tested on auto-encoded cohort spectra have demonstrated greater than 89% accuracy for assigning the parity statuses of wild *An. arabiensis* and *An. gambiae* s.s. [25], while age determination of 94% accuracy for three age classes has been achieved for wild-caught *Anopheles coluzzii*, *An. arabiensis*, and *An. gambiae* using MIRS and deep transfer learning modeling [26]. The additional spectral processing and more complex modeling required for high accuracy age determination suggests that subtle variations in wild cohort spectra and age structure may occur even over brief time periods, resulting in the unsuitability of using regression modeling alone as a chemometric.

A more direct approach than precise age grading for wild mosquito populations, as suggested by Joy et al. [24], is a form of monitoring through tracking changes in cohort age structure over time. This approach is highly applicable to assessments of control efficacy through the knowledge of population age patterns over pre- and postadulticiding periods, leading to a better understanding of the timing of recruitment. Cohort age trending information supplied rapidly and cost-effectively would enable control personnel to fine-tune the type, number, and timing of treatments for problematic mosquito species in areas of concern. A monitoring approach is supported by analysis of shortwave infrared spectra from laboratory reared female *An. gambiae* s.l. and *Ae. aegypti*, which exhibit higher outlier fractions for young (< 7 days) versus old (≥ 7 days) cohorts [27], and by shortwave infrared spectroscopic (SWIRS) investigation of wild host-seeking *Coquillettidia perturbans* collected over different dates and seasons at several sites in central Massachusetts, USA, which show variation in the fraction of spectra outliers, implying that changes in cohort age structure occur throughout the summer season [27].

We tested the possibility that adulticiding efficacy could be measured by using a monitoring approach through comparison of pre- and post-treatment SWIR spectra outlier fractions for wild cohorts of mosquitoes of different species. Higher fraction post-treatment would indicate older mosquitoes being killed and replaced by younger ones, thereby demonstrating effective treatment and reduction of a portion of the population associated with the greatest risk for transmission (i.e., older mosquitoes). Lower outlier fraction post-treatment would imply recruitment of older mosquitoes into the treatment area and/or low control efficacy, while fractions of roughly equal value pre- and post-treatment would show that insecticide application had little impact. Pre- and postcontrol trend evaluation in outlier fraction would inform control efforts regarding problematic species that may need additional treatment applications. Also, the evaluation of patterns of abundance versus outlier fraction by species would assist in determining temporal patterns in the emergence of adults from new cohorts of mosquitoes and recruitment during the post-treatment periods. The outlier fraction technique has additional benefits in that it uses economical equipment, is more rapid than ovary dissection, entails minimal training, and does not require machine learning [27], all characteristics that allow for ease and flexibility of integration into mosquito surveillance and control operations, especially those in areas with limited available resources.

## Methods

### Mosquito collection and scanning totals

**Fig. 1:**
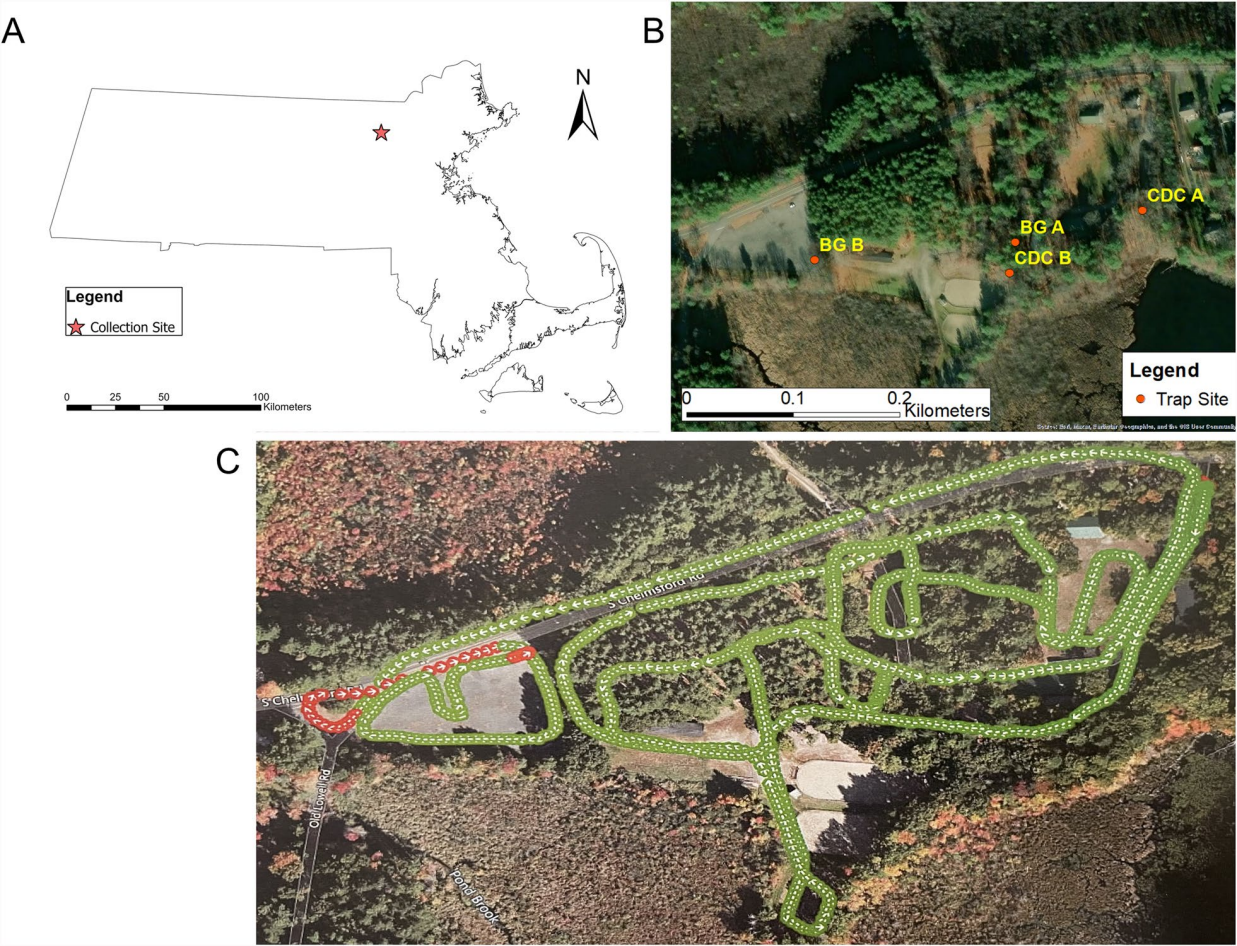
Treatment site details. **A** Collection site location within the state of Massachusetts, USA; **B** Trap type and site locations, **C** Treatment log data, the green arrows indicate the pump is active

Mosquito collection was conducted during July and August 2023 at a rural site near Westford, Massachusetts (Fig. 1A) with surrounding habitat consisting of wooded areas, human infrastructure, wetlands, waterbodies (pond and stream), and open fields all within a one-kilometer radius of trap sites. Mosquito traps consisted of two Centers for Disease Control and Prevention miniature light traps with CO_2_ as an attractant and two BG-Sentinel traps (Biogents USA, Moorefield, VA) (Fig. 1B). Collections were made before and after four bi-weekly ULV adulticide treatments with Zenivex E4 (pyrethroid derivative, Etofenprox) performed via truck-mounted spraying with a flow rate of 4.5 floz/min (Fig. 1C). The traps were set in the field and each operated for 24 h (10:00–10:00) before insecticide application (pre-ULV adulticide application) and for 48 h (10:00–10:00) after treatment (post application).

The following descriptive terms are used for mosquito collection dates: PRE is 1 day pretreatment, POST d1 is 1 day post-treatment, POST d2 is 2 days post treatment, and POST (d1 + d2) is aggregated post-treatment days (Additional file 1: Supplementary Table 1). Mosquitoes were killed immediately after collection by freezing at −40 °C and were preserved at −15 °C in capped Eppendorf centrifuge tubes until scanning. The species included in this study were *Aedes cinereus* Meigen, *Aedes vexans* (Meigen), *Anopheles punctipennis* (Say), *Anopheles quadrimaculatus* Say, *Coquillettidea perturbans* (Walker), *Culiseta melanura* (Coquillett), a complex of *Culex pipiens* Linnaeus and *Culex restuans* Theobald, *Ochlerotatus abserratus* (Felt and Young), *Ochlerotatus canadensis* (Theobald), *Ochlerotatus excrucians* (Walker), and *Psorophora ferox* (von Humboldt).

Treatments were aggregated to produce a minimum of 100 PRE, 100 POST d1, and 100 POST d2 specimens for each species. If fewer than 100 specimens were collected for aggregated collection dates, all specimens collected and not damaged by trapping were scanned. PRE, POST d1, and POST d2 trap collections were allocated proportionally to the final totals so that collections from every trap and collection date for each species were represented in scanning samples (Table 1).

**Table 1:** Aggregated species abundance and scanning totals

		POST		
	PRE	d1	d2	Total
Abundance				
* Ae. cinereus*	369	302	264	935
* Ae. Vexans*	74	63	88	225
* An. punctipennis*	119	101	95	315
* An. quadrimaculatus* s.l.	23	16	16	55
* Cq. perturbans*	1011	795	1179	2985
* Cs. melanura*	177	108	182	467
* Cx. pipiens/restuans* complex	2391	1723	2570	6684
* Oc. abserratus*	35	23	22	80
* Oc. canadensis*	1274	810	794	2878
* Oc. excrucians*	36	16	10	62
* Ps. ferox*	239	70	126	435
Scanned				
* Ae. cinereus*	178	125	118	421
* Ae. vexans*	66	52	87	205
* An. punctipennis*	105	86	85	276
* An. quadrimaculatus* s.l	15	15	13	43
* Cq. perturbans*	121	124	119	364
* Cs. melanura*	116	109	134	359
* Cx. pipiens/restuans* complex	320	117	115	552
* Oc. abserratus*	34	19	21	74
* Oc. canadensis*	189	117	115	421
* Oc. excrucians*	30	9	9	48
* Ps. ferox*	147	69	121	337

### Scanning setup, technique, timeframe, and conditions

**Fig. 2:**
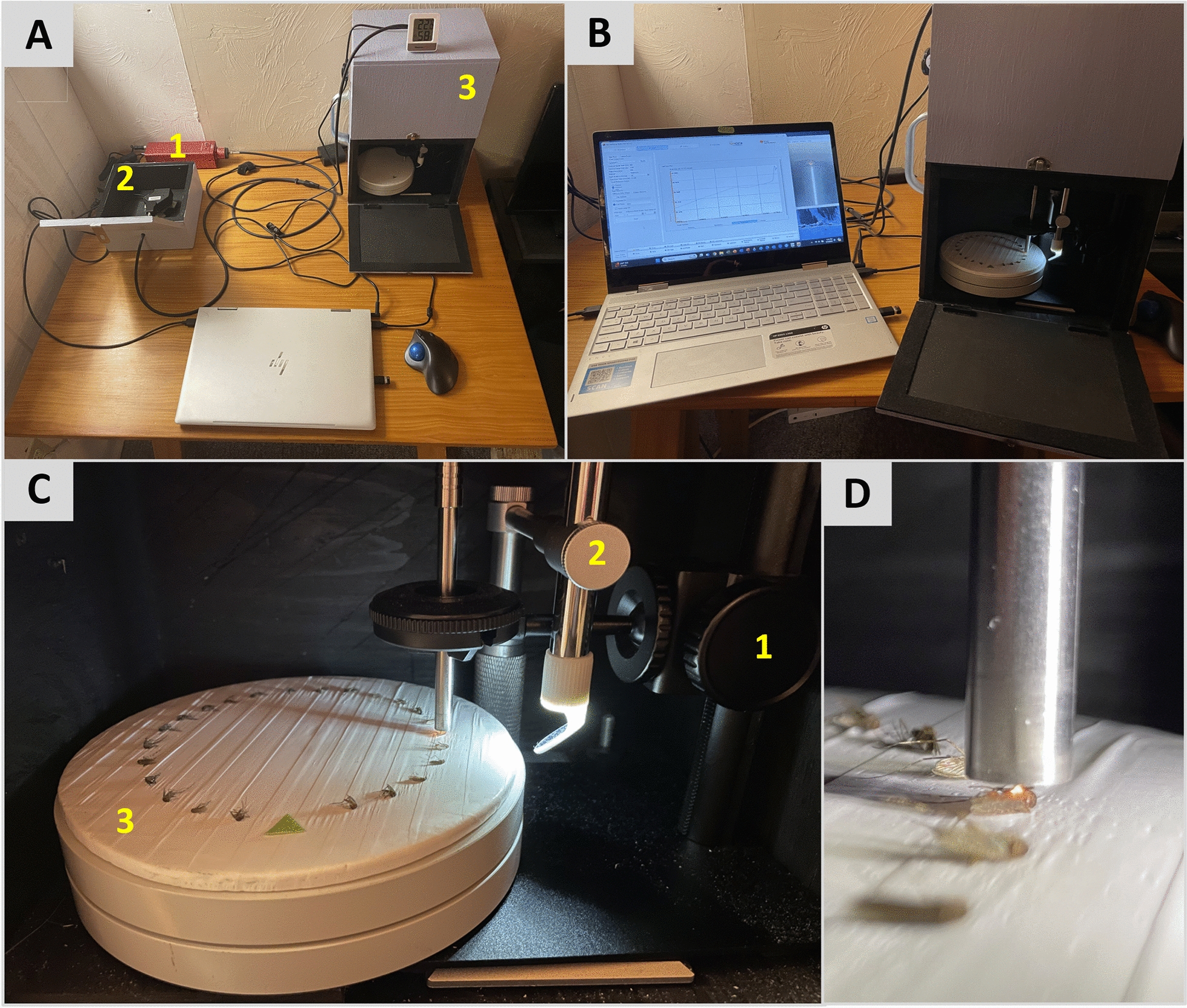
Scanning apparatus and setup. **A** (1) Light source, (2) spectrometer within cabinetry, (3) scanning cabinetry; **B** scanning software and USB microscope video (left), mosquitoes positioned for scanning (right); **C** scanning apparatus detail: (1) adjustable precision microscope stand with probe held in chuck, (2) USB microscope with right-angle mirror (note: USB microscope LED lighting is turned on but was turned off and all cabinet doors closed prior to scanning), (3) cork disk wrapped in Teflon tape (reflectance standard) on rotating stand with mosquitoes positioned for scanning; **D** detail showing probe head adjusted to within 1 mm of the mosquito cuticle

Scanning setup and technique were as described in Swab et al. [27] (Fig. 2), with one exception: A digital USB microscope (Shenzhen Andonstar Tech Co. Ltd, Shenzhen, China) was added to improve focusing consistency (Fig. 2B), as video from the microscope ensured that the probe head was consistently focused to within 1 mm of the mosquito cuticle without contacting the cuticle surface (Fig. 2C, D). Mosquitoes were analyzed as soon as possible after the summer field season. The average length of time in storage varied from 131 days (4 months) for *Oc. abserratus* to 247 days (7.5 months) for *An. quadrimaculatus* s.l., with an average storage time of 6 months for all species (Table 2). Ambient relative humidity (RH) and temperature were recorded during scanning with a digital hygrometer (Thermopro TP49, Lawrenceville, GA, USA) placed atop scanning cabinetry, with ambient conditions averaging 31.9% RH and 21.9 °C throughout the scanning period (Table 2).

**Table 2:** Average preservation time and ambient scanning conditions

Species	Preservation time		Scanning conditions	
	Days	Months	Relative Humidity (RH) (%)	Temperature (*T*) (°C)
*Oc. abserratus*	131	4.3	32.9	22.0
*Oc. canadensis*	143	4.7	34.2	21.9
*Ae. cinereus*	170	5.6	31.5	21.8
*Cx. pipiens/restuans* complex	180	5.9	31.0	22.0
*Ps. ferox*	193	6.3	28.9	21.5
*Oc. excrucians*	198	6.5	29.6	21.8
*Cs. melanura*	203	6.6	33.2	22.6
*Cq. perturbans*	229	7.5	32.4	21.9
*Ae. vexans*	229	7.5	30.6	21.7
*An. punctipennis*	242	7.9	32.6	22.2
*An. quadrimaculatus* s.l	247	8.1	34.4	21.8
	–	–	–	–
Minimum (MIN)	131	4.3	28.9	21.5
Maxiumum (MAX)	247	8.1	34.4	22.6
Average (AVG)	197	6.5	31.9	21.9

### Data analysis, handling, and measurement tolerance

Data analysis followed methodologies detailed in Swab et al. [27]. In brief, each spectrum was preprocessed using the Standard Normal Variate scatter-corrective method, spectrums were then combined by cohort and collection date, cohort spectra outliers were calculated following the 1.5 interquartile range (IQR) rule, and cohort spectra outlier fraction (*f*) was calculated through Eq. 1.

1 $${\text{Cohort Spectra Outlier Fraction }}\left( f \right) \, = \, \sum {\text{ spectra outlier points}}/\sum {\text{ spectra points}}$$

The resolution of the spectrometer used is 3.5 nm, generating 228 SWIR absorbance measurements per scan, resulting in a total of 1,413,600 records requiring 0.08 GB digital storage for this study. Microsoft Access (v. 2108) and Excel (v. 2108) were used for data storage, handling, and analysis. Two scanning trials of aspirin tablets (i.e., standardized control) were conducted after mosquito scanning was completed to measure the inaccuracy of the scanning technique and calculate a measurement tolerance. Aspirin tablets were used as a standard based upon technical documentation and recommendation from the manufacturer of the spectrometer [28].

## Results

### Species *f* and differences in *f*

Based on aspirin standards, scanning measurement tolerance for *f* was estimated to be 0.0008 when rounded to four significant digits, deemed stringent enough for the scope of the study (Additional File 2: Supplementary Table 2). Species *f* for PRE, POST d1, POST d2, and POST (d1 + d2, combined post-treatment) collection dates fell outside of measurement tolerance (Table 3), while actual differences in species *f* for consecutive collection dates were also beyond tolerance except for the difference between PRE and POST d1 for *Oc. excrucians* (Table 4), indicating no change in *f* between PRE and POST d1. Relative differences in *f* for consecutive collection days showed a range of 0 to nearly 20 depending upon species (Table 5).

**Table 3:** POST belongs over d1, d2, and (d1 + d2)

Species		POST		
	PRE	d1	d2	(d1 + d2)
*Ae. cinereus*	0.0240	0.0641	0.0262	0.0559
*Ae. vexans*	0.0163	0.0438	0.0267	0.0347
*An. punctipennis*	0.1019	0.0644	0.0502	0.0588
*An. quadrimaculatus* s.l	0.0513	0.0127	0.0626	0.0482
*Cq. perturbans*	0.0078	0.0165	0.0120	0.0149
*Cs. melanura*	0.0187	0.0651	0.0315	0.0465
*Cx. pipiens/restuans* complex	0.0031	0.0612	0.0690	0.0666
*Oc. abserratus*	0.0106	0.0673	0.0174	0.0419
*Oc. canadensis*	0.0024	0.0046	0.0033	0.0038
*Oc. excrucians*	0.0018	0.0022	0.0261	0.0033
*Ps. ferox*	0.0951	0.0942	0.0624	0.0914

**Table 4:** Actual differences in species *f* for consecutive collection dates

Species	POST d1-PRE	POST d2-POST d1
*Ae. cinereus*	0.0401	−0.0379
*Ae. vexans*	0.0275	−0.0171
*An. punctipennis*	−0.0376	−0.0142
*An. quadrimaculatus* s.l	−0.0386	0.0499
*Cq. perturbans*	0.0087	− 0.0045
*Cs. melanura*	0.0464	−0.0337
*Cx. pipiens/restuans* complex	0.0581	0.0078
*Oc. abserratus*	0.0567	−0.0499
*Oc. canadensis*	0.0022	−0.0013
*Oc. excrucians*	*0.0004*	0.0239
*Ps. ferox*	−0.0009	−0.0318

The italicized value is less than the tolerance shown in Additional File 2: Supplementary Table 2

**Table 5:** Relative differences in species *f* for consecutive collection dates

Species	POST d1/PRE	POST d2/POST d1
*Ae. cinereus*	2.7	0.4
*Ae. vexans*	2.7	0.6
*An. punctipennis*	0.6	0.8
*An. quadrimaculatus* s.l	0.2	4.9
*Cq. perturbans*	2.1	0.7
*Cs. melanura*	3.5	0.5
*Cx. pipiens/restuans* complex	19.8	1.1
*Oc. abserratus*	6.4	0.3
*Oc. canadensis*	1.9	0.7
*Oc. excrucians*	0	11.9
*Ps. ferox*	1.0	0.7

### Linear regressions: independent variables versus *f* and differences in *f*

**Fig. 3:**
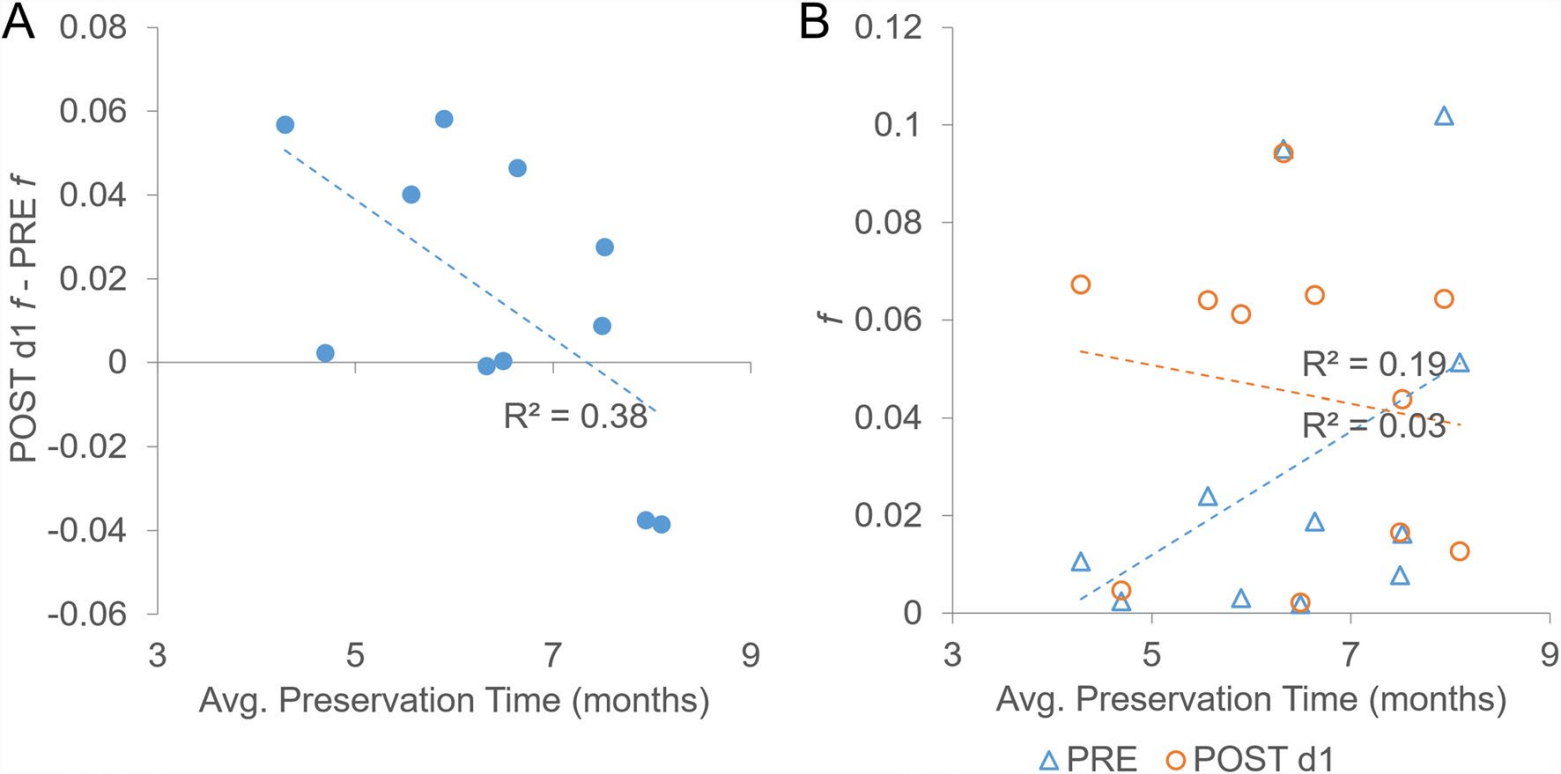
Preservation time versus actual difference in POST d1 *f* and PRE *f*. **A** Preservation time versus POST d1* f*—PRE* f*; **B** Preservation time versus POST d1 *f* and PRE *f*

Linear regressions show no relationships between the independent variables of abundance, number of specimens scanned, average preservation time, average scanning ambient relative humidity, and average scanning ambient temperature versus *f* (Table 6). No relationships are shown for linear regressions for these same variables versus the actual differences in *f* other than a negative correlation for preservation time and POST d1 *f*–PRE *f* (Table 7), while no relationships are shown between variables and relative differences in *f* (Table 8). The negative correlation between preservation time and actual difference in PRE versus POST d1 *f* indicates that as species preservation time increases, PRE *f* increases while POST d1 *f* decreases. However, the relationship is weak to moderate at best, as can be seen by the low coefficient of determination for the correlation (Fig. 3A) and lack of linear correlations in the source data (Fig. 3B).

**Table 6:** Series of linear regressions examining abundance, number scanned, preservation time, and ambient scanning RH and *T* on *f*

Predictor variable	Coefficient	Standard error (SE)	*P* value
PRE *f*			
Abundance	−2 × 10^−5^	2 × 10^−5^	0.24
#Scanned	−5 × 10^−5^	1 × 10^−4^	0.70
Preservation time (mos.)	0.013	0.009	0.18
RH (%)	−0.003	0.007	0.67
T (°C)	−0.013	0.042	0.77
POST d1 *f*			
Abundance	−5 × 10^−6^	2 × 10^−5^	0.79
#Scanned	1 × 10^−4^	2 × 10^−4^	0.58
Preservation time (mos.)	−0.004	0.008	0.64
RH (%)	− 0.006	0.005	0.28
* T* (°C)	0.012	0.036	0.75
POST d2 *f*			
Abundance	6 × 10^−6^	9 × 10^−6^	0.55
#Scanned	−2 × 10^−5^	2 × 10^−4^	0.91
Preservation time (mos.)	0.007	0.005	0.22
RH (%)	−0.003	0.004	0.43
T (°C)	−0.006	0.026	0.82

Confidence level of 95%, degrees of freedom (d.f.) of 9

**Table 7:** Series of linear regressions examining abundance, number scanned, preservation time, and ambient scanning RH and *T* on actual differences in *f*

Predictor variable	POST d1 *f*—PRE *f*			POST d2 *f*—POST d1* f*		
	Coefficient	SE	*P* value	Coefficient	SE	*P* value
Abundance	−5 × 10^−5^	5 × 10^−5^	0.38	2 × 10^−5^	4 × 10^−5^	0.58
#Scanned	− 2 × 10^−4^	2 × 10^−4^	0.25	− 5 × 10^−4^	5 × 10^−4^	0.28
Preservation time (mos.)	− *0.017*	*0.007*	*0.04*	0.011	0.007	0.14
RH (%)	−0.003	0.006	0.62	0.003	0.005	0.60
*T* (°C)	0.025	0.039	0.54	−0.018	0.034	0.61

Confidence level of 95%, d.f. of 9

**Table 8:** Series of linear regressions examining abundance, number scanned, preservation time, and ambient scanning RH and *T* on relative differences in *f*

	POST d1 *f*/PRE *f*			POST d2 *f*/POST d1 *f*		
Predictor variable	Coefficient	SE	*P* value	Coefficient	SE	*P* value
Abundance	5.2	10.6	0.64	−4.9	2.6	0.09
#Scanned	−8.9	6.9	0.23	−3.0	3.8	0.44
Preservation time (mos.)	−1.5	1.4	0.32	0.5	0.9	0.58
RH (%)	−0.4	1.0	0.72	−0.5	0.6	0.45
*T* (°C)	3.5	6.4	0.60	−2.5	4.0	0.55

Confidence level of 95%, df of 9

### Trends in *f* and *f* versus abundance

**Fig. 4:**
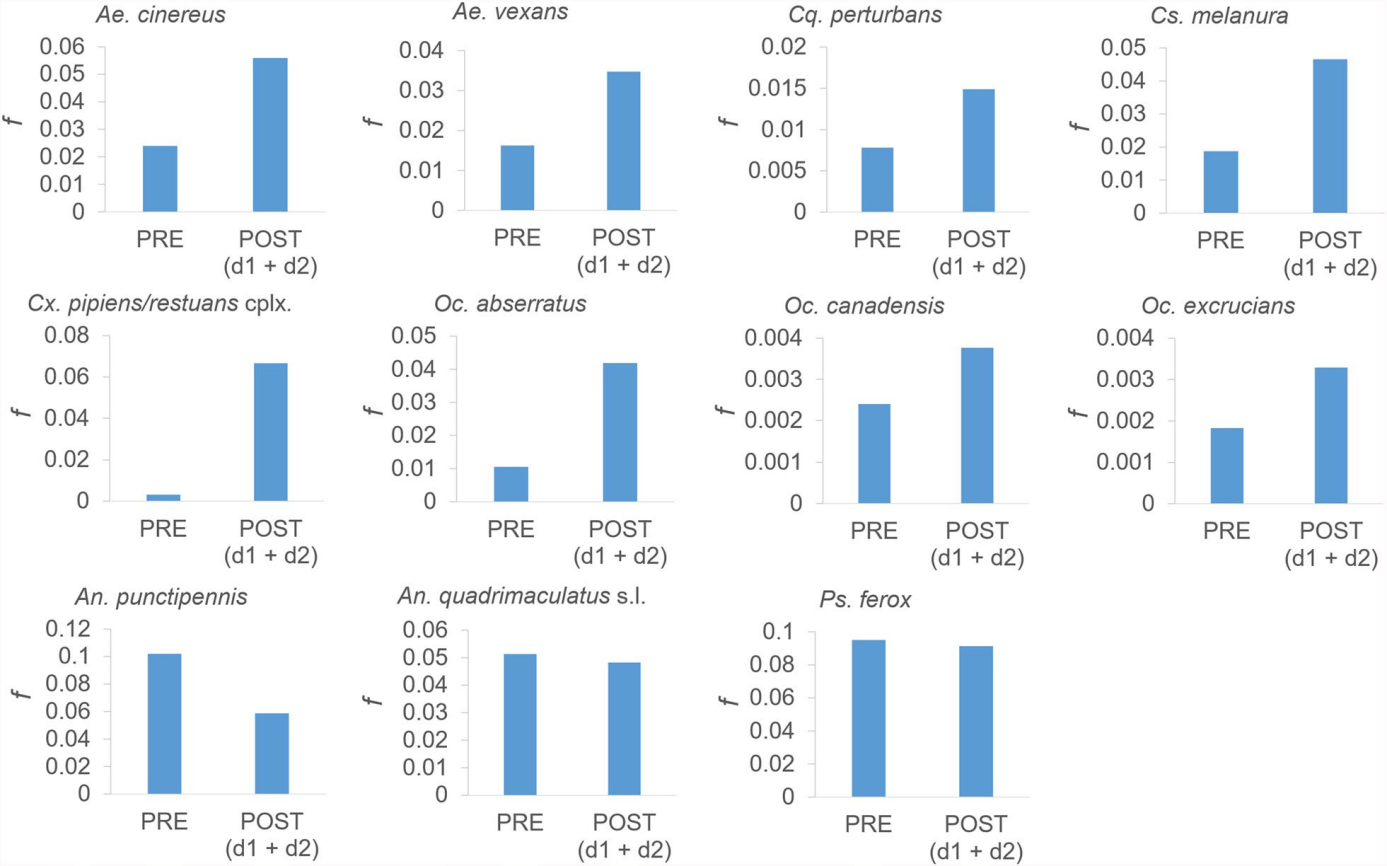
PRE *f* versus POST (d1 + d2) *f* by species

A total of eight out of ten species (*Cq. perturbans*, *Cs. melanura*, *Cx. pipiens/restuans*, *Aedes* sp, and *Ochlerotatus* sp.) display an increase in *f* from pretreatment compared with aggregated post-treatment days (Fig. 4, top and middle), suggesting that the combined post-treatment cohorts consist of younger mosquitoes by weighted average age as compared with the pretreatment cohort, and the younger mosquitoes occurred at sufficiently high levels over the post-treatment period to increase *f* beyond PRE values. The remaining three species (*Ps. ferox* and *Anopheles* spp.) showed a reduction in *f* for the post-treatment period (Fig. 4, bottom), implying that combined post-treatment cohorts were composed of older mosquitoes by weighted average age as compared with pretreatment cohorts.

Although abundance is not indicative of cohort age structure, comparison of abundance to *f* assists in determining whether changes in abundance are caused by the impact of younger or older mosquitoes. Abundance was reduced from PRE to POST d1 for all species except *Oc. excrucians* (Table 1) demonstrating that ULV adulticiding had an impact on those species, attributable to a reduction in a portion of the population. However, change in abundance from POST d1 to POST d2 varied by species, with some species returning to pretreatment levels while others never fully rebound (Table 1), implying that recovery is species specific. Mosquito collection was accomplished with traps designed for capture of host-seeking mosquitoes, therefore emergences of gravid mosquitoes sequestered within the study area were not measured and post treatment abundance changes were caused by recruitment.

**Fig. 5:**
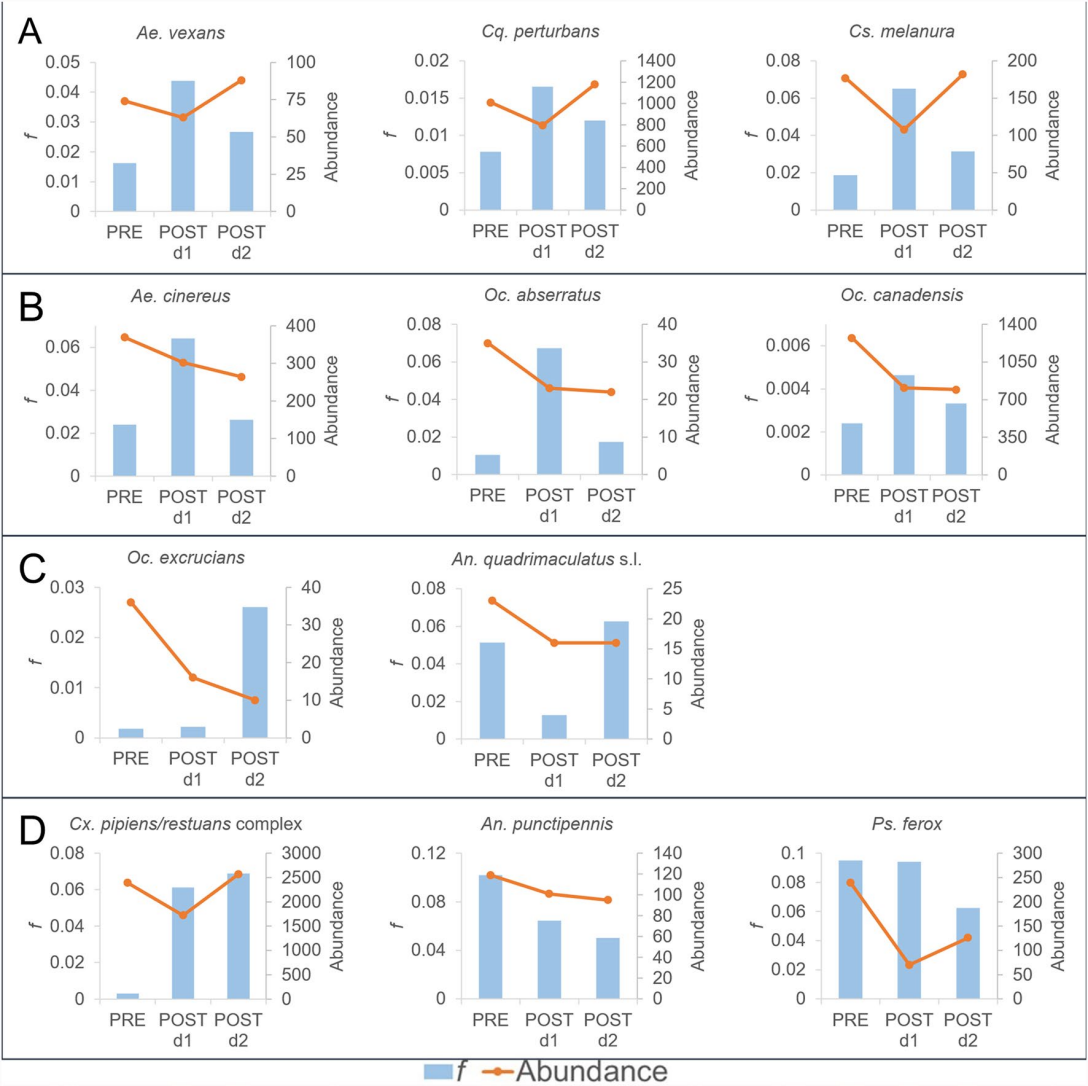
Classification of trends in abundance and *f* by species. **A** Recruitment of younger mosquitoes does not offset abundance reduction by POST d1, while recruitment of older mosquitoes offsets the abundance reduction by POST d2; **B** Recruitment of younger mosquitoes does not offset abundance reduction by POST d1, while recruitment of older mosquitoes does not offset abundance reduction by POST d2; **C** Recruitment of younger mosquitoes on POST d2 does not offset abundance reduction by POST d2; **D** Species that do not fit into A-C, including the *Cx. pipiens/restuans* complex for which recruitment of younger mosquitoes on POST d1 and POST d2 offsets the abundance reduction by POST d2, *An. punctipennis* for which recruitment of older mosquitoes on POST d1 and POST d2 does not offset abundance reduction by POST d2, and *Ps. ferox* for which recruitment of older mosquitoes on POST d2 does not offset abundance reduction by POST d2

Analysis of differences in abundance and *f* by species over consecutive collection days indicates that the variation in cohort age structure over the post-treatment period is species-specific and likely driven by recruitment over the collection period, and species may be categorized into four groups based on trends in abundance and *f*. In group A (Fig. 5A), which includes *Ae. vexans*, *Cq. perturbans*, *Cs. melanura*, recruitment of younger mosquitoes does not offset abundance reduction from control by POST d1, while recruitment of older mosquitoes offsets the abundance reduction by POST d2. Group B (Fig. 5B) is composed of *Ae. cinereus*, *Oc. abserratus*, and *Oc. canadensis*, for which recruitment of younger mosquitoes does not offset abundance reduction from control by POST d1, while recruitment of older mosquitoes does not offset abundance reduction by POST d2. Group C (Fig. 5C) contains *Oc. excrucians* and *An. quadrimaculatus* s.l. and shows that recruitment of younger mosquitoes on POST d2 does not offset abundance reduction from control by POST d2. Group D (Fig. 5D) consists of species that do not fit into groups A–C: the *Cx. pipiens/restuans* complex for which recruitment of younger mosquitoes on POST d1 and POST d2 offsets the abundance reduction from control by POST d2; *An. punctipennis* for which recruitment of older mosquitoes on POST d1 and POST d2 does not offset abundance reduction from control by POST d2; and *Ps. ferox* for which recruitment of older mosquitoes on POST d2 does not offset abundance reduction from control by POST d2.

## Discussion

Regardless of whether POST d1 and d2 are combined or kept separate (e.g., increasing temporal granularity), the same eight species still show higher POST *f* values as compared with PRE *f* (group A, group B, *Oc. excrucians*, and the *Cx. pipiens/restuans* complex), while *An. punctipennis* and *Ps. ferox* demonstrate consistently lower POST* f* than PRE *f*, and *An. quadrimaculatus* s.l. shows the highest *f* on POST d2. Considering that abundances were reduced post-treatment for the *Anopheles* spp. and *Ps. ferox* (Table 1), this implies that the SWIRS method may be unsuitable for these species, or that aspects of their phenology led to lower levels of younger mosquitoes in post-treatment populations that were not enough to increase *f* over PRE values. Alternatively, the significant delay in scanning the *Anopheles* spp. (270 days) may have rendered the method insensitive.

All species in the study, with the possible exceptions of *Oc. abserratus* and *Oc. excrucians* exhibited different patterns of abundance from July through August at an untreated site in Connecticut [29]; as such, it is likely that differences in phenology would produce variable patterns in *f* over the post treatment period. Aspects impacting species phenology that could influence POST d1 and POST d2 cohort age structure include voltinism, egg hatch delay (asynchronous development), dispersal rate and range, longevity, and age-susceptibility to insecticides. While voltinism of New England mosquito species is well understood, and hatch delay is linked to larval habitat temperatures, dispersal ranges and longevity in the wild have not been thoroughly researched for all species included here.

**Fig. 6:**
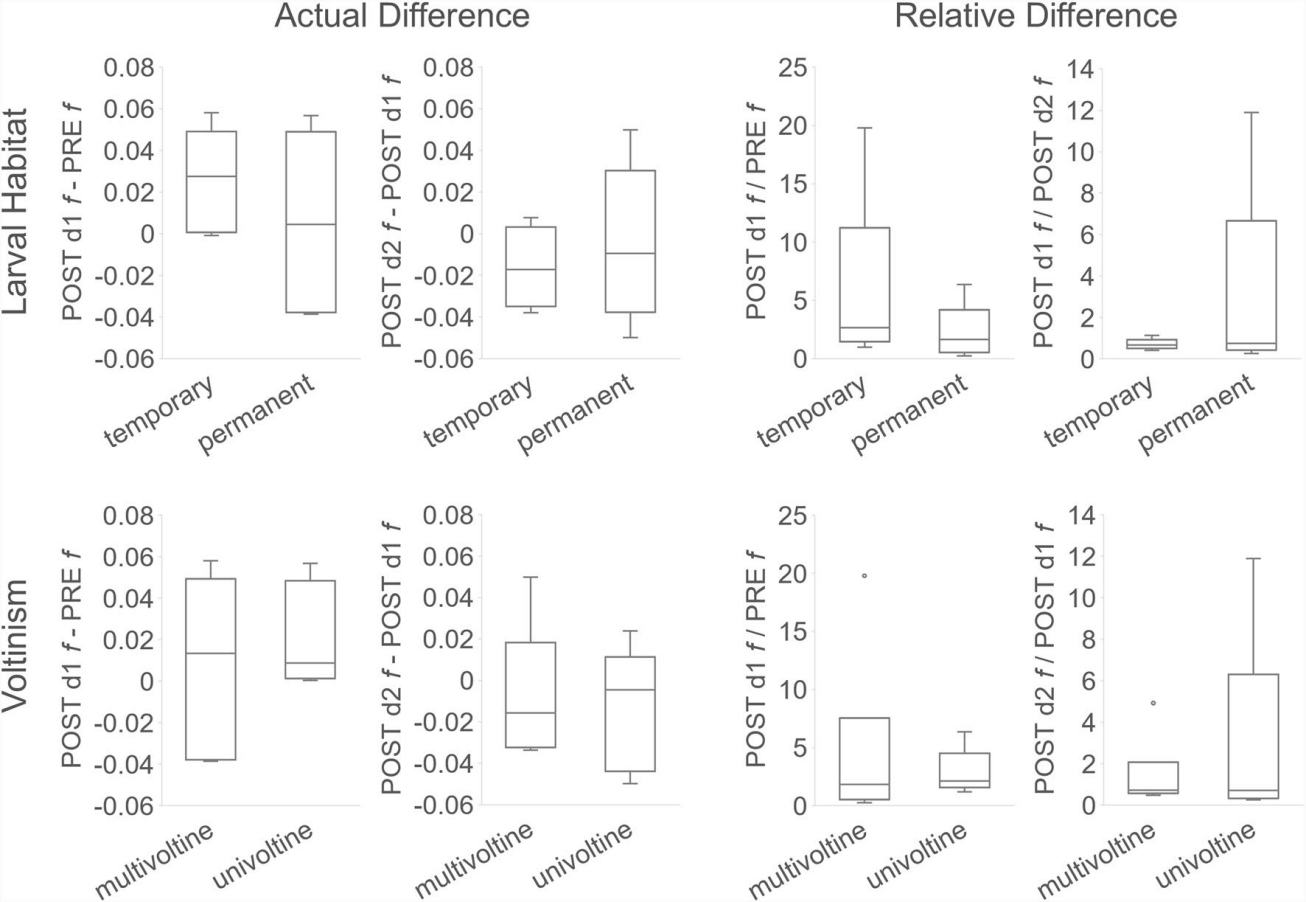
Distribution of species voltinism and larval habitat type versus difference in *f* over consecutive collection dates. Larval habitat is equal to the water type

Hatch delay occurs in eggs that are resistant to desiccation where larval habitat is temporary and susceptible to drying and reflooding, and it may occur for both univoltine and multivoltine species in Massachusetts [30 Chapter IV]. The classification groups described previously display different mixes of species larval habitat and voltinism in the New England region (Table 9); however, no distribution patterns for differences in *f* over consecutive collection days are evident for either aspect (Fig. 6). This may be due in part to weak statistical power caused by small sample sizes, with *N* = 5 or 6 for all species.

**Table 9:** Species larval habitat, voltinism, and maximum dispersal range

Group	Species	Larval habitat water type^a^	Voltinism^a^	Average maximum flight distance (km)^b^
A	*Ae. vexans*	Temporary	Multivoltine	5.73
A	*Cq. perturbans*	Permanent	Univoltine	3.40
A	*Cs. melanura*	Permanent	Multivoltine	9.80
B	*Ae. cinereus*	Temporary	Univoltine	1.60
B	*Oc. abserratus*	Permanent	Univoltine	
B	*Oc. canadensis*	Temporary	Univoltine	
C	*An. quadrimaculatus* s.l	Permanent	Multivoltine	3.42
C	*Oc. excrucians*	Permanent	Univoltine	10.00
D	*An. punctipennis*	Permanent	Multivoltine	16.90
D	*Cx. pipiens/restuans* complex	Temporary	Multivoltine	9.70
D	*Ps. ferox*	Temporary	Multivoltine	2.50

^a^Andreadis et al.[52]

^b^Verdenschot and Lototskaya [53]

Dispersal rate, in part, would impact recruitment timing, and daily dispersal rates for species in this study that have been investigated include *Ae. vexans* [31], *Cx. pipiens pallens* [32], *An. quadrimaculatus* [33], and *Cq. perturbans* [34], all of which exhibited daily rates larger than the treatment area of approximately 0.5 km^2^ (Fig. 1). As such, it seems reasonable to assume that population recruitment from either newly emerged or older mosquitoes could occur by POST d1 or d2 for the genera represented in the prior research. More complete data exist for average maximum flight range for all but two species in the study (Table 9), but the data show no relationships to differences in *f* over consecutive collection days by species (Table 10). However, maximum flight range is not representative of dispersal rate, and therefore, the lack of correlation may be due in part to the relatively brief 2-day post-treatment collection period and a small sample size.

**Table 10:** Linear regressions performed on average maximum flight distance^a^ and differences in* f*

Response variable	Coefficient	SE	*P*-value
POST d1 *f*—PRE *f*	−0.001	0.003	0.68
POST d2 *f*—POST d1* f*	0.0003	0.002	0.89
POST d1 *f*/PRE *f*	0.193	0.463	0.69
POST d2 *f*/POST d1 *f*	0.108	0.287	0.72

Confidence level of 95%, df of 8

^a^From Table 9

The impact of longevity on post-treatment population abundance and age structure is difficult to deduce since life spans in the wild for the species included here have not been thoroughly researched. Specimens of *Ae. vexans* have been found to live 113 days [35], while *Ae. canadensis* has been described as “very long-lived” compared with other species [35]. Lambert et al. [36] used a meta-analysis to estimate the average survival times for unfed, laboratory reared mosquitoes and calculated that *Aedes* genera are longest-lived at 8 days, followed by *Anopheles* at 7 days and *Culex* at 3 days. However, nourished wild mosquitoes may live much longer as postcapture longevity of wild *Cx. pipiens* has been found to average between 50 and 70 days for July and August in Greece, with variations in longevity depending on capture date [37]. A large relative difference between POST d1 *f* and PRE *f* for a species (Table 5) denotes older cohorts prior to treatment, and since cohort data are aggregated over July and August, this suggests relatively mature populations throughout the 2-month period and greater longevity than a species with low relative difference between POST d1 *f* and PRE *f*. However, Anderson et al. [29] in a study of mosquito species collected at a site in Connecticut found that *Cx. pipiens* and *Cx. restuans*, species that comprise the complex that exhibits the greatest relative difference between POST d1 *f* and PRE *f* (Table 5), show significant variability in abundance over July and August, suggesting that “young” versus “old” is likely a shifting scale depending on species. As such, inferring species longevity from differences in PRE *f* and POST *f* for interpretation of how it may be driving post-treatment trending is beyond the scope of this study due to low temporal granularity caused by aggregating results of four seasonal treatments into one sample.

**Fig. 7:**
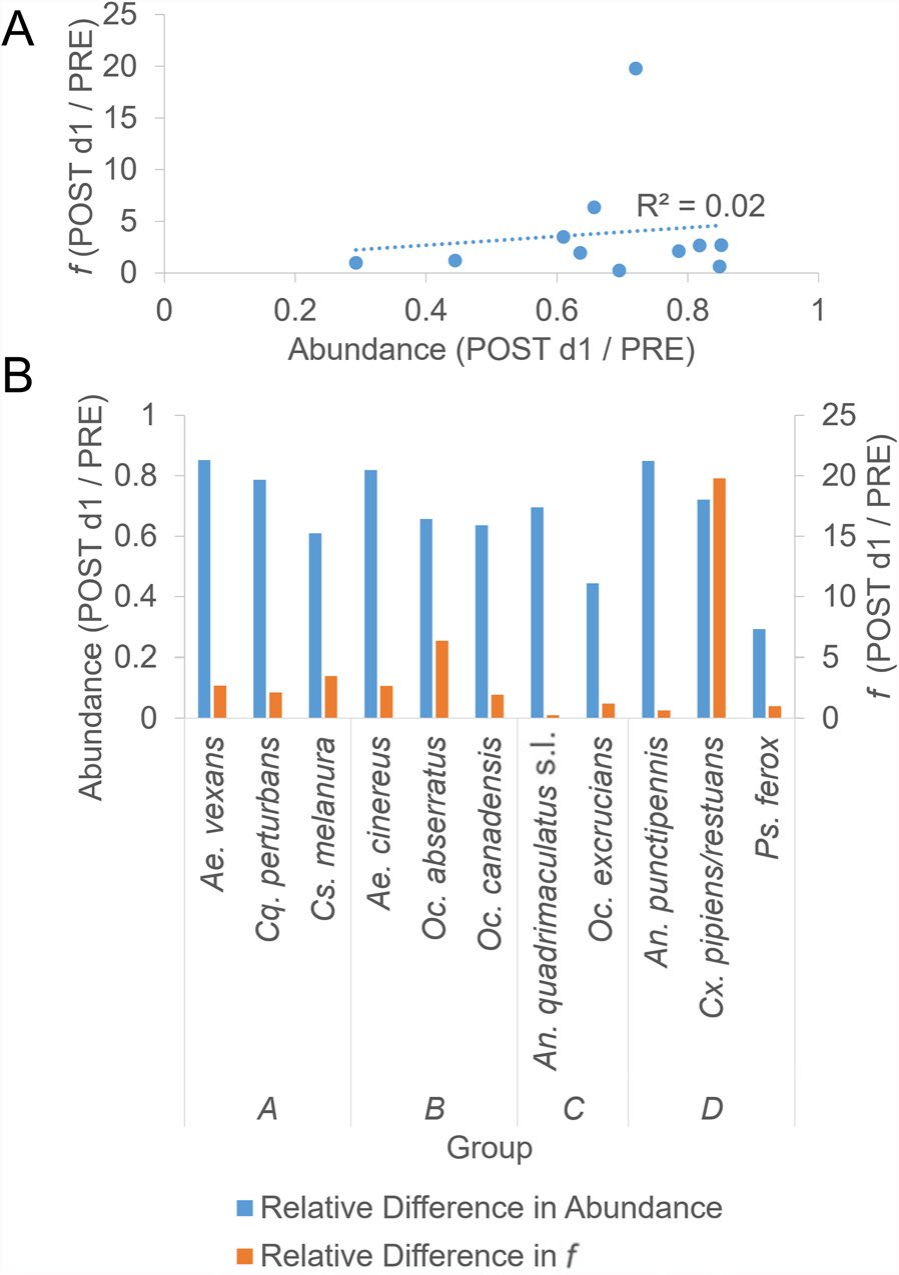
Relative difference in POST d1 and PRE collection dates for abundance versus *f*. **A** Relative difference in abundance versus *f*; **B** relative difference by group classification shown in Fig. 5

Age susceptibility to insecticides would affect post-treatment cohort age structure and has been shown for adult *Ae. aegypti* [38] *An. gambiae* [38, 39], and *Culex quinquefasciatus* [40], making it likely that other genera and species may show similar age-related changes in susceptibility to insecticides. Since the insecticidal ingredient in Etofenprox has a half-life of 6.2 h in air [41] (p. 27), age susceptibility to Zenivex would be most apparent in the relative difference for abundance versus *f* over PRE and POST d1. Greater susceptibility would be indicated by larger relative abundance reduction occurring for those species that have the lowest PRE *f* relative to POST d1 *f*. However, there is no correlation between these variables (Fig. 7A), and no visual indication that relative change in abundance and *f* corresponds to the group classification (Fig. 7B).

Other factors that impact adult cuticle and therefore possibly influencing SWIR spectra include larval diet and environment, adult host preference, and adult infection with pathogens and parasites. Research on *An. albimanus* indicates that larval and adult diet as well as *Plasmodium* infection of adults all affect adult cuticular hydrocarbons [42], while larval environment influences adult cuticular surface microbiota [43]. Moreover, blood from different host species has been shown to impact the cuticular lipids of *Ae. aegypti* [44], while the presence of both *Wolbachia* and Zika virus infection of *Ae. aegypti* has been demonstrated by NIRS [12, 45]. However, Liebman et al. [11] found that NIRS and regression modeling predictive ability was 80–90% for binary age classification of *Ae. aegypti* despite larval and adult diet variation, with the best performing model trained on larvae with the most varied diet, while NIRS characterization of *Plasmodium* infected *An. gambiae* and *An. stephensi* has met with only mixed success [46–48]. Due to the conflicting nature of results, future investigation should be directed toward determining the impacts of diet, environment (e.g., mosquito vectors from distinct geographic locations, local microbiota), and parasitic infection on SWIR spectra and *f*.

Lag between collection and scanning dates resulted in preservation times between 4.3 and 8.1 months under freezing conditions (Table 2), and ice crystals were observed to have formed inside some of the capped centrifuge storage tubes prior to scanning indicating possible specimen moisture loss. Desiccation can impact absorbance measurements since the O–H bond in water results in absorbance peaks at 1210 nm and 1450 nm within the SWIRS range [49]. Reduction in area under the 1450 nm peak has been shown to occur with decreasing moisture content for a specimen of *An. gambiae* [19], while concentration coefficients for emulsified lipid and water mixtures have been estimated with > 95% accuracy in the SWIR range using a fiber optic probe coupled with a portable spectrometer [50]. However, refrigeration of up to 2 months has been shown to have little impact on the accuracy of age prediction models developed from NIRS spectra for laboratory reared *An. gambiae* [7]. Considering the preservation times of between 4 and 8 months for this study that resulted in ice crystal formation in some storage tubes and may have contributed to the weak correlation between preservation time and actual difference between POST d1 *f* and PRE *f*, as well as impacting *Anopheles* spp. results, and it is recommended that future work incorporate cold storage preservation times of less than 2 months.

Future research should also concentrate on problematic mosquitoes of importance that are either sentinel species or suspected bridge vectors of pathogens infecting human and animal health. Sampling plans that increase temporal granularity to four to five control treatments with post-treatment periods of 5–6 days, an approach modeled after Lopez et al. [3], should be incorporated for better understanding of post-treatment recruitment. We also suggest recording collection site ambient temperature and relative humidity to account for meteorological impact and that specimens be frozen for no longer than 2 months prior to scanning. A control group from an adjacent untreated site (i.e., sham control) should be incorporated, and a statistical power analysis conducted to determine a minimum sample size. The accuracy of the SWIRS technique as compared with classical ovary dissections has not been established, and future work is planned using field collections to verify that age trends as determined by SWIRS *f* track those estimated from dissections.

The ultimate (future) objective of SWIRS research is to provide a tool that will aid in managing mosquitoes by enabling personnel to assess control efficacy and set action thresholds through monitoring changes in age structure during control treatments. The approximate turnaround time for calculating *f* from a sample of 50 mosquitoes is 2 h for the tasks of specimen preparation, scanning, and data handling. As such, the method has the capability to inform real-time decision making in treatment strategies and compares favorably to a dissection rate of ~16 mosquitoes over 2 h using Detinova ovarian tracheation for parity determination [51]. However, further study is needed to ascertain if turnaround time can be reduced through smaller sample size (as determined through statistical power analysis), eliminating the repeat scanning for each specimen, and incorporating improvements to the method such as a modified tablet autosampler. Though the technique has good potential for integration into control programs, the feasibility for field deployment is low due to the need for specimens to be consistently positioned and motionless under the scanning probe head. Thus, the current platform technology is most appropriate for use in a laboratory environment.

SWIRS research has the potential for helping to set control action thresholds through routine surveillance via monitoring changes in age structure at localized “hot spots,” where mosquito pools have tested positive for pathogens. Changes in age structure over time at locations of concern would apprise personnel of mosquito population age-progression and inform strategies as to the need and timing of treatment. Moreover, site specific monitoring would be of interest to medical entomologists since correlating age-trends with environmental conditions, pathogen extrinsic incubation periods, host life cycles, and surveillance data would help to refine the understanding of the ecology of mosquito-borne diseases.

The resources needed for control programs to adopt SWIRS include a cost of approximately $3600 USD for equipment [27] plus a laptop, encompassing a footprint of less than one square meter (Fig. 2). Training in specimen preparation, software, scanning technique, and data handling and analysis is straightforward and is estimated to take as little as 1 h. The low resolution of the spectrometer in our study (228 measurements per scan) allowed Microsoft products to be used for data handling and analysis; however, incorporating a higher-resolution spectrometer generating additional measurements per scan would require a more powerful data analysis program such as R for calculating *f* values. R is an open-source programming language for statistical computing, data analysis, and data visualization, which is widely used in data science. Future work includes development and publication of a Standard Operating Procedure for efficiency and consistency in method application.

## Conclusions

This study shows that changes in SWIR cohort spectra outlier fraction *f* occur between pre- and post-ULV adulticide treatment for wild cohorts of eleven species of mosquitoes. Eight species demonstrate higher *f* post treatment, suggesting younger mosquitoes replacing older ones that have been killed by treatment, while the remaining three species exhibit an opposite trend. Further examination of abundance versus *f* by species and collection day suggests recruitment of mosquitoes into the treatment area over the post-treatment period, information that would prove beneficial to control personnel. Despite unanswered questions, we believe that the SWIRS technique demonstrates promise for monitoring the relative age structure of wild mosquito populations, evaluating mosquito control efficacy, and meeting a key requirement for assessing vectorial capacity.

## Supplementary Information

Additional file 1.

Additional file 2.

## Data Availability

Absorbance data generated for and used in the study, along with a read me file and code tables are located at GitHub, https://github.com/CSwab01/Mosquito.

## Abbreviations

EIP: Extrinsic incubation period
IQR: Interquartile range
IRS: Infrared spectroscopy
LED: Light emitting diode
NIRS: Near-infrared spectroscopy
MIRS: Mid-infrared spectroscopy
SWIR: Shortwave infrared
SWIRS: Shortwave infrared spectroscopy
ULV: Ultra-low volume
USB: Universal serial bus
