# Supplementary material for: Age structure of cohorts of mosquitoes from the field using shortwave infrared spectroscopy before and after ULV adulticide treatment
Source: Parasit Vectors. 2025 Jul 1;18:250. doi: 10.1186/s13071-025-06873-1 (PMC12220619; doi:10.1186/s13071-025-06873-1)
Supplement: Supplementary file 1 — Additional file 1. [file 13071_2025_6873_MOESM1_ESM.docx]

**Additional File 1: Table S1** Treatment and collection dates

|  | Collection Dates | |  |
| --- | --- | --- | --- |
| Treatment Date | PRE | POST d1 | POST d2 |
| 7/12/2023 | 7/11/23 | 7/13/23 | 7/14/23 |
| 7/26/2023 | 7/25/23 | 7/27/23 | 7/28/23 |
| 8/9/2023 | 8/8/23 | 8/10/23 | 8/11/23 |
| 8/23/2023 | 8/22/23 | 8/24/23 | 8/25/23 |
