# Supplementary material for: Age structure of cohorts of mosquitoes from the field using shortwave infrared spectroscopy before and after ULV adulticide treatment
Source: Parasit Vectors. 2025 Jul 1;18:250. doi: 10.1186/s13071-025-06873-1 (PMC12220619; doi:10.1186/s13071-025-06873-1)
Supplement: Supplementary file 2 — Additional file 2. [file 13071_2025_6873_MOESM2_ESM.docx]

**Additional File 2: Table S2** Estimated measurement tolerance

|  |  | Scanning Details | |  |  |
| --- | --- | --- | --- | --- | --- |
| CVS^a^ aspirin tablets | N | Date | Count |  | *f* |
| Expiration date: 2/25 | 100 | 5/3/2024 | 200 |  | 0.0098 |
| Expiration date: 9/25 | 100 | 5/9/2024 | 200 |  | 0.0106 |
|  |  |  |  |  | ---- |
| Measurement tolerance^b^ |  |  |  |  | 0.0008 |

^a^CVS Pharmacy Inc., Woonsocket, RI

^b^Measurement tolerance (estimated error) = difference between *f* values
